# 28. Autoimmune limbic encephalitis in a patient with lupus

**DOI:** 10.1093/rap/rky033.019

**Published:** 2018-09-20

**Authors:** Mohammad Solaiman, Yusuf Patel

**Affiliations:** Rheumatology, Hull Royal Infirmary, Hull, UNITED KINGDOM

**Introduction:** We present an interesting case of autoimmune limbic encephalitis with associated VGKC antibodies and MRI scan abnormalities.

**Case description:** A 44 year old female first presented to us at the age of 33 in 2004 with arthralgia, rash, raynaud’s and positive ANA (anti-ds-DNA 166, chromatin, Lo and La) and was commenced on hydroxychloroquine. She progressed well until 2013 when she developed rheumatoid pattern arthritis/synovitis and required methotrexate (ACPA/RF negative) and a short course of low dose oral steroid. Due to resistant arthritis despite combination MTX and hydroxychloroquine, we progressed on to rituximab therapy. Unfortunately she developed angioedema after the first infusion. However, her symptoms improved transiently with rituximab, but later flared requiring repeat steroid. Due to persistent arthritis, we switched therapy to MMF and then added abatacept in subsequent months. The arthritis responded well to abatacept therapy. She was admitted to hospital in December 2015 with breathlessness, leg swelling, syncope, skin rash and vasculitis. CT confirmed pulmonary embolus, bi basal pneumonia, small pericardial effusion, bilateral pleural effusion, and also R leg DVT, Echo- PAP 61 but right heat catheter did not revealed PAH. The CT-TAP showed low volume lymphadenopathy but no malignancy. Abatacept was discontinued transiently and restarted for arthritis after three months, although we questioned adherence to therapy and switched to intravenous abatacept. Despite this she required intermittent and then regular steroids to suppress disease. The patient chose to terminate abatacept in May 2016. She was then admitted in August 2016 for increasing confusion, headache and blurring of vision. There were no skin/joint and systemic symptoms. Initially she was apyrexial and examination was unremarkable. Her CRP was raised at 36, anaemia, LDH 654, ferritin 2405, urine leuc 3+, nitrates positive. She received empirical therapy for a urinary tract infection. Serial blood culture showed protieus/enterococcus/mirabilas and antibiotics were adjusted accordingly. The patient remained happily confused despite therapy for infection. CT head was normal but MRI showed T2 signal in right temporo-occipital lobe. Cerebrospinal fluid showed opening pressure 6mmhg, no organisms, glucose 3.3, raised protein 0.92. Blood test demonstrated Ds-DNA >300, negative virology, negative ANCA and lupus anticoagulant/anti-cardiolipin/anti-B2 GP, but Voltage-gated-potassium channel antibody VGKC IgG was raised at 241u/L (0 to 69). NMDA-N, Musk IgG Ab, Ach receptor Ab were all negative. In conjunction with the neurologist, it was decided that the clinical picture was consistent with autoimmune limbic encephalitis, and steroid therapy was escalated to IV methylprednisolone followed by high dose oral steroid. PET-CT did not show any vasculitis or malignancy. Despite steroid and antibiotic therapy on the intensive care unit, she developed recurrent pyrexia, variable CRP, hypoxia, fluctuating consciousness and progressive deterioration. DNAR was discussed with the family and a decision was made for ward based care. Unfortunately she had progressive deterioration and died in November 2016.

**Discussion:** This patient had an initial good response to therapy for lupus, but subsequently needed biologics and steroids to suppress arthritis/vasculitis. Despite withdrawal of biologics and treatment for infection, she developed progressive neurological problems with features of autoimmune limbic encephalitis. Steroid escalation was not successful in controlling this manifestation and subsequent infection/pressure sores became a problem to manage with an unfortunate final outcome. This case highlights the difficulty in managing immunosuppression in the presence of infection, and also provides characteristic features of a very rare neurological manifestation of autoimmune disease

**Key Learning Points:** Consider infection but also autoimmune limbic encephalitis in new onset confusion in lupus patients.

**Disclosure: M. Solaiman:** None. **Y. Patel:** None.

